# Exploring the accuracy of self-reported maternal and newborn care in select studies from low and middle-income country settings: do respondent and facility characteristics affect measurement?

**DOI:** 10.1186/s12884-023-05755-7

**Published:** 2023-06-16

**Authors:** Katharine J. McCarthy, Ann K. Blanc, Charlotte E. Warren, Ashish Bajracharya, Ben Bellows

**Affiliations:** 1 Department of Population Health Science & Policy, Icahn School of Medicine at Mount Sinai, New York, NY USA; 2 Blavatnik Women’s Health Research Institute, New York, NY USA; 3 Population Council, New York, USA; 4 Population Council, Phnom Penh, Cambodia; 5 Nivi, Inc. Sudbury, Massachusetts, USA

**Keywords:** Validation, Maternal and newborn care, Postnatal care, Antenatal care, Meta-analysis of diagnostic test accuracy, Intervention coverage, Monitoring

## Abstract

**Background:**

Accurate data on the receipt of essential maternal and newborn health interventions is necessary to interpret and address gaps in effective coverage. Validation results of commonly used content and quality of care indicators routinely implemented in international survey programs vary across settings. We assessed how respondent and facility characteristics influenced the accuracy of women’s recall of interventions received in the antenatal and postnatal periods.

**Methods:**

We synthesized reporting accuracy using data from a known sample of validation studies conducted in Sub-Saharan Africa and Southeast Asia, which assessed the validity of women’s self-report of received antenatal care (ANC) (*N* = 3 studies, 3,169 participants) and postnatal care (PNC) (*N* = 5 studies, 2,462 participants) compared to direct observation. For each study, indicator sensitivity and specificity are presented with 95% confidence intervals. Univariate fixed effects and bivariate random effects models were used to examine whether respondent characteristics (e.g., age group, parity, education level), facility quality, or intervention coverage level influenced the accuracy of women’s recall of whether interventions were received.

**Results:**

Intervention coverage was associated with reporting accuracy across studies for the majority (9 of 12) of PNC indicators. Increasing intervention coverage was associated with poorer specificity for 8 indicators and improved sensitivity for 6 indicators. Reporting accuracy for ANC or PNC indicators did not consistently differ by any other respondent or facility characteristic.

**Conclusions:**

High intervention coverage may contribute to higher false positive reporting (poorer specificity) among women who receive facility-based maternal and newborn care while low intervention coverage may contribute to false negative reporting (lower sensitivity). While replication in other country and facility settings is warranted, results suggest that monitoring efforts should consider the context of care when interpreting national estimates of intervention coverage.

**Supplementary Information:**

The online version contains supplementary material available at 10.1186/s12884-023-05755-7.

## Introduction

The vast majority of maternal and newborn deaths occur in settings characterized by the least amount of data on intervention coverage and quality of care [1, 2]. Accurate data on effective intervention coverage, the proportion of individuals experiencing health gains from a service among those who need the service, is key to monitoring and scaling up the delivery of essential interventions to populations in need [3, 4]. Intervention coverage data is routinely used to track progress in national and global commitments such as Sustainable Development Goal 3—which includes a target to reduce the maternal mortality ratio to less than 70 per 100,000 live births, Countdown to 2030, as well as WHO strategies Ending Preventable Maternal Mortality (EPMM) and the Every Newborn Action Plan (ENAPP) [5–8]. In response to evidence that intervention coverage indicators may overestimate progress due to poor content of care[9–12], strategies have shifted emphasis from monitoring health care access to quality adjusted coverage [4, 13].

**Fig. 1:**
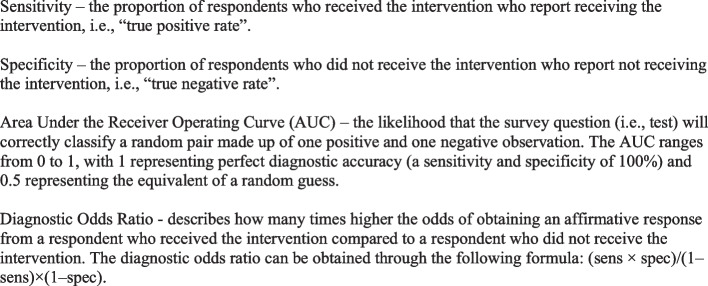
Indicator Key Terms

In resource-limited settings, data on the coverage of maternal and newborn health interventions often relies on women’s reports collected in nationally representative household surveys such as the Demographic and Health Surveys (DHS) and Multiple Indicator Cluster Surveys (MICS) [14]. Self-reported data from population-based surveys, however, assumes that women accurately recall interventions received during the antenatal, intrapartum, and postnatal periods. A growing number of studies have assessed the validity of self-reported maternal and newborn care interventions used (or with the potential to be used) in these surveys [15–22]. Collectively, evidence from these studies demonstrate considerable variability in indicator validity across settings (accuracy metrics defined in Fig. 1), leading to the question of why? [15–17, 19, 23].

The validity of self-reported data on maternal and newborn health interventions received may be influenced by a variety of factors. These include women not knowing whether an intervention occurred because they were not aware it was performed (i.e., it was not explained, or in the case of newborn interventions, it was performed outside of the mother’s view). Recall may also be influenced by the nature and timing of questions. Prior research on maternal recall of interventions received in the intrapartum period has found that indicators which include technical terms (e.g., names of medications or diseases), refer to the timing (e.g., whether an intervention occurred immediately or within the first few minutes after birth) or sequence of events (e.g., whether infant wrapped before being laid on the mother’s chest) or are performed within the first hour of birth were unlikely to be recalled with high accuracy [15–17, 24]. The recall period may also influence reporting. Both DHS and MICS surveys typically ask women to recall events related to births occurring 2–5 years prior. Previous analysis of the recall accuracy of intrapartum and immediate postnatal care in Kenya has suggested that while accuracy generally declines with time, select interventions that are recalled with high accuracy at facility discharge maintain acceptable accuracy at 13 to 15 months follow-up [17].

Question comprehension related to respondent background characteristics or their expectation of care may also influence recall accuracy. For example, if a woman had a positive experience and/or delivered in a facility perceived to be high quality, she may be more likely to indicate that an intervention assumed to be beneficial occurred. Background characteristics may influence reporting if lower education contributes to poor understanding of questions with technical or complex wording or if higher parity leads to confusion of care with a previous pregnancy. Adolescents may have lower reporting accuracy because they have had less familiarity with the health system. Accurate coverage estimates among adolescents is of particular importance given that infants born to nulliparous adolescent mothers are at higher risk of neonatal and infant mortality than any other maternal age group, are more likely to delay care seeking, and receive fewer components of maternal health care [25–28], which further emphasizes the need for accurate coverage estimates in this group.

Explanatory analyses to examine patterns in the accuracy or consistency of reporting by respondent characteristics (e.g., age, education or prior parity) or by infant or facility characteristics have varied by indicator and setting, making it difficult to discern broad patterns [18, 29–31]. One study in rural Nepal found that maternal age and place of delivery (facility vs. home) did not influence maternal reporting of infant outcomes; while accuracy related to infant birth size was higher among multiparous mothers [30]. Another study of intrapartum care recall among mothers in Ethiopia found that older women (ages 35–39 relative to ages 10–24) were more likely to report postpartum complications inconsistently while those who delivered in a health facility were more likely to inconsistently report on newborn immediate thermal care practices [29]. A third study which assessed recall of facility-based postnatal care interventions among women in Kenya, found no pattern in reporting accuracy by maternal age, education, parity or infant age [18]. Overall, heterogeneity in the types of indicators assessed, study methodology, question wording and limited sample sizes for subgroup analysis in some studies complicates collective understanding.

To better inform how respondent and facility characteristics that influence the accuracy of self-reported maternal and newborn care, we synthesized data from five previous validation studies conducted in low and middle-income country settings. Studies were purposely sampled due to known similarities in question wording and validation design. Using these data, we examine whether respondent characteristics (e.g., age, education, prior parity), facility quality, or intervention coverage consistently predicted recall accuracy.

## Methods

### Data sources

We synthesize patterns in reporting accuracy from a unique set of known validation studies led by the Population Council which used the same validation design to assess comparable indicators of maternal and newborn care in multiple low and middle-income country settings. We draw on five validation studies of maternal and newborn care reported across two publications [18, 32]. Three studies assessed antenatal care indicators (Bangladesh, Cambodia, Kenya) and five studies (Bangladesh, Cambodia, Kenya (2) and eSwatini) assessed postnatal care indicators for the mother and newborn. Studies were purposely selected from two multi-country intervention studies as each study used the same or very similar wording for client questionnaires and observer checklists (Additional Files 1 and 2). Table 1 describes the study context and sample characteristics for each study. In all studies the samples consisted of women of reproductive age who received facility-based care and were interviewed at discharge (exit interview). Women’s self-reports at exit interview were compared against direct observation by a trained third-party observer using a structured checklist (reference standard).

**Table 1:** Study characteristics

Country, Validation Study	Type of care	Study context [facility type and number]	Participant characteristics [Sample size]
Bangladesh, Voucher study, McCarthy et al., 2020 [32]	Antenatal care and postnatal care (between 24 h and 8 weeks of birth)	Data collected in 2011 and 2013 in 22 upazila health complex (UHC) facilities in sub-districts from six divisions: Barisal, Chittagong, Dhaka, Khulna, Rajshahi and Sylhet. The majority (77) of facilities offered comprehensive obstetric care while about one-quarter offered basic obstetric care (24). 17 provided referral to a district hospital or maternal and child welfare center	*N* = 1036 for antenatal care clients; *N* = 208 for postnatal care Matched observations and exit interviews from women aged 18 to 45 years old who received ANC or PNC for themselves (as well as their newborns)
Cambodia, Voucher study McCarthy et al., 2020 [32]	Antenatal care and postnatal care (between 24 h and 8 weeks of birth)	Data collected in 2010 and 2012 in 40 government health facilities from 8 provinces: Kampong Thom, Kampot, Prey Veng, Kampong Cham, Kep, Siem Reap, Svay Reing, and Oddor Mean Chey. All but two health facilities were health centers; two were former district hospitals	*N* = 952 antenatal care clients, *N* = 635 postnatal clients Matched observations and exit interviews from women aged 18 to 45 years old who received ANC or PNC for themselves (as well as their newborns)
Kenya, Voucher study McCarthy et al., 2020 [32]	Antenatal care and postnatal care (between 24 h and 8 weeks of birth)	Data collected in 2010 and 2012 in 62 health facilities located in Kisumu, Kiambu, Kitui counties and the Korogocho and Viwandani informal settlements in Nairobi. Facilities were either public (*N* = 40), private-for-profit (*N* = 10), faith-based (*N* = 9) or NGO (*N* = 3). The majority of facilities were hospitals (60.9), followed by health centers (30.8), and nursing homes, dispensaries or clinics (8.3)	*N* = 1,176 antenatal clients, *N* = 1,619 postnatal clients Matched observations and exit interviews from women aged 18 to 45 years old who received ANC or PNC for themselves (as well as their newborns)
Kenya, Integra Study, McCarthy et al., 2018 [18]	Routine postnatal care for mother or newborn (24 h – 10 weeks following birth)	Data collected in 2009, 2011 and 2012 from 12 facilities (4 hospitals and 8 health centers) from Central and Eastern provinces	545 postnatal care clients Women ages 15 to 45 who attended a postnatal check for herself and/or newborn (> 24 h – 10 weeks of birth) at a participating study facility were eligible for inclusion
eSwatini, Integra Study, McCarthy et al., 2018 [18]	Routine postnatal care for mother or newborn (24 h – 10 weeks following birth)	Data collected in 2009, 2011 and 2012 from 8 facilities (public health units/ MCH-FP) in Lubombo, Manzini and Shiselweni regions	319 postnatal care clients Women ages 15 to 45 who attended a postnatal check for herself and/or newborn (> 24 h – 10 weeks of birth) at a participating study facility were eligible for inclusion

Data on routine postnatal care in Kenya and eSwatini were drawn from the Integra Initiative, a quasi-experimental study which aimed to strengthen provider capacity to give postnatal care to the (1) infant and (2) the mother, integrated with (3) family planning, (4) HIV counseling, testing and services, and (5) screening for and management of sexually transmitted infections [33]. The study population for the Integra study was women who attended a postnatal check for themselves and/or for their newborn (> 24 h to < 10 weeks of delivery) at a participating study facility and who provided informed consent to be interviewed. There were eight facilities (public health units/MCH-FP) in three regions (Lubombo, Manzini and Shiselweni) of eSwatini and 12 public health facilities located in the former Eastern province (present-day Kitui and Makueni counties) in Kenya. In total, matched exit interview and observer data were available for 545 women in Kenya and 319 in eSwatini.

Data on receiving antenatal or postnatal care in Bangladesh, Cambodia, and Kenya were originally collected as part of an evaluation of a voucher and accreditation intervention (henceforth “voucher study”) which assessed whether the voucher program improved service quality by verifying service delivery through reimbursements to providers [34–36]. The theory of change was that subsidized service demand stimulates greater service utilization and competition between service providers to improve service quality [37]. Providers were effectively rewarded for quality service delivery through reimbursement of service provision at a contracted level of quality. As such, voucher intervention facilities were used as a proxy for higher quality of care relative to propensity-score matched control facilities. Although voucher intervention status is not a comprehensive measure of facility quality, existing evidence supports the link between such voucher-accreditation approaches with improved facility readiness and quality for reproductive health service delivery [37, 38]. While the influence of voucher schemes on antenatal care has been comparatively less studied, evaluation of the Kenya Safe Motherhood Voucher Scheme found significant improvement in the overall quality in the components of delivered postnatal care relative to comparable control facilities [39]. Evaluation of the Bangladesh voucher scheme also found some evidence of postnatal care service quality improvements among high performing voucher facilities relative to control areas, however, differences in antenatal service quality were less substantial [36]. While the Cambodia scheme was found to increase ANC service utilization, no published findings with regard to quality are available [40].

Voucher studies in this analysis included a total of 22 government health facilities from six divisions of Bangladesh (Barisal, Chittagong, Dhaka, Khulna, Rajshahi and Sylhet), 40 government facilities from five provinces of Cambodia (Kampong Speu, Kampong Thom, Kampot, Prey Veng, Takeo), and 62 facilities in Kenya, which were a mixture of public (64%), private-for-profit (16%), faith-based (15%) or NGO (5%) and were located in Kisumu, Kiambu, Kitui counties and two informal settlements in Nairobi. Approximately half of facilities in each location were assigned to voucher or propensity-matched control facility status. In total, 3,169 women were interviewed and observed for antenatal care (*n* = 1,036 in Bangladesh, 957 in Cambodia and 1,176 in Kenya) and 2,462 for postnatal care (*n* = 208 in Bangladesh, 635 in Cambodia and 1,619 in Kenya).

### Indicator selection and data extraction

All comparable indicators with available validation data from at least three studies were extracted, as this was considered sufficient for meta-analysis [41]. For each indicator, two-by-two contingency tables which compared women’s self-report to the observer report (reference standard) were tabulated to obtain the number of true positive, false positive, false negative and true negative responses. “Don’t Know” responses were set to missing for validity analysis but reported in the tables as this response type is distinct from women who think they know whether an intervention was received.

### Predictors

Predictors were defined a priori as maternal age, maternal education, parity, type of facility (whether facilities were voucher accredited or control) and intervention coverage (observed intervention prevalence in each setting). Predictor selection was informed by prior evidence of factors with the potential to influence reporting accuracy. Age strata were adolescent (ages 15 to 20) vs. adult (ages 21–52). The adolescent age group was inclusive of clients aged 20 to maximize sample size for stratification. Prior parity was defined as first pregnancy (for ANC) or birth (PNC) vs. two or more prior pregnancies or births. Education was defined as less than primary completion vs. primary completion or greater. As described above, whether facilities were voucher accredited was used as a proxy for facility quality. Finally, intervention coverage was calculated as the mean observed indicator prevalence in each study.

### Analysis

To examine differences in reporting accuracy by respondent and facility characteristics, forest plots of sensitivity (true positive rate) and specificity (true negative rate) stratified by predictors of interest were examined. As a summary benchmark, high sensitivity and specificity was considered 80% or higher. This threshold was selected based on the empirical distribution of the accuracy of self-reported data related to maternal and newborn care. Stratified forest plots for age are shown, as this was the primary outcome of interest. To statistically test whether predictors were a source of heterogeneity between primary validation studies we used fixed effects and bivariate random effects models, as data allowed.

Bivariate random effects models were constructed when study indicators were validated in at least five studies, the minimum number required for model estimation [41, 42]. A bivariate random effects approach is the standard in diagnostic test accuracy as both sensitivity and specificity are simultaneously estimated, which accounts for the trade-off between sensitivity and specificity [43]. Typically, as a threshold is varied to increase the sensitivity, the specificity often decreases and vice versa [44]. Bivariate models also account for variation in the diagnostic threshold used across studies (e.g., differences in observer ratings due to variation in training procedures or other factors across studies). Bivariate models accommodate study-aggregate covariates (i.e., intervention coverage, i.e., the prevalence of a given indicator in each study) to examine whether the predictor affects sensitivity, specificity, or both. Intervention coverage was examined as a predictor for PNC reporting accuracy only, as a minimum of five studies was required for parameter estimation. Within-study predictors (i.e., individual-level respondent and facility characteristics) predictors are not accommodated in bivariate random effects models and were compared by assessing the degree of overlap in summary estimates (and corresponding 95% CIs) for stratified bivariate models.

As all ANC indicators as well as the predictor facility quality were collected in three studies only, univariate fixed effects models were constructed. Univariate models estimate the diagnostic odds ratio, which describes the odds of obtaining an affirmative response from a respondent who received the intervention compared to a respondent who did not receive the intervention [45]. To assess whether results varied by level of the predictor, overlap in the summary diagnostic odds ratio (DOR) and corresponding 95% CIs for fixed effects models were examined. Univariate fixed effects models do not account for the trade-off between sensitivity and specificity or between-study heterogeneity [41, 42], however, they give reasonably consistent estimates of the DOR irrespective of variation in diagnostic threshold [46]. Given these limitations, the ANC and facility-quality results from univariate fixed effects models are presented in Additional files 3, 4 and 5. Emphasis is given to results from bivariate fixed effects models in the discussion of study results.

Finally, indicators based on a small number of true (observed) positive or true negative cases which resulted in low precision (margin of error greater than 15 percentage points for bivariate models or a diagnostic OR of five or greater for univariate models) are reported in the data tables, but not discussed in the text. Results from the bivariate and univariate models were obtained using the *mada* package in R Studio (Version 1.1.383, Boston MA) [44].

## Results

### Study and sample descriptions

Participant sociodemographic characteristics across studies are presented in Table 2. The pooled sample size for postnatal care was 3,326 women and for antenatal care indicators was 3,169 women. There were comparable indicators with sufficient sample size (no multiple zero cells) in three or more countries for 12 postnatal care indicators and six antenatal indicators.

**Table 2:** Sample descriptive statistics

	Voucher Study			Integra Study	
**Postnatal care (PNC) clients**	Bangladesh *N* = 208 n (%)	Cambodia *N* = 635) n (%)	Kenya *N* = 1,619 n (%)	eSwatini *N* = 319 n (%)	Kenya *n* = 545 N (%)
**Age (Mean, SE)**	23.8 (0.33)	26.8 (0.21)	25.8 (0.15)	26.3 (0.25)	25.1 (0.32)
**Age group**					
15 to 20	67 (32.2)	56 (8.8)	309 (19.1)	71 (22.3)	87 (16.0)
21 to 29	109 (52.4)	390 (61.4)	900 (55.6)	171 (53.6)	307 (56.3)
30 to 39	32 (15.4)	174 (27.4)	377 (23.3)	74 (23.2)	139 (25.5)
40 +	0 (0.0)	15 (2.4)	32 (2.0)	3 (0.9)	12 (2.2)
**Education**					
Less than primary	45 (21.4)	77 (12.2)	104 (6.4)	24 (7.5)	217 (39.8)
Primary completion	55 (26.4)	306 (48.2)	913 (56.4)	59 (18.5)	224 (41.1)
Secondary	104 (50.0)	203 (31.9)	416 (25.7)	109 (34.3)	77 (14.1)
Tertiary	7 (3.4)	49 (7.7)	186 (11.5)	126 (39.6)	27 (5.0)
**Number of times given birth**					
One	88 (42.1)	251 (39.6)	531 (32.8)	109 (34.2)	163 (29.9)
Two	66 (31.6)	190 (29.9)	436 (26.9)	81 (25.3)	126 (23.2)
Three	27 (13.2)	101 (15.9)	295 (18.2)	66 (20.6)	105 (19.3)
Four or more	27 (13.2)	93 (14.6)	359 (22.2)	63 (19.9)	151 (27.7)
**Marital status**					
Never married	0 (0.0)	3 (0.5)	189 (11.7)	176 (55.3)	63 (11.6)
Ever married	208 (100.0)	632 (99.5)	1430 (88.3)	143 (44.7)	482 (88.4)
**Antenatal care (ANC) clients**	Bangladesh *N* = 1,036 n (%)	Cambodia *N* = 957 n (%)	Kenya *N* = 1,176 n (%)		
** Age (Mean, SE)**	23.5 (0.15)	27.8 (0.13)	25.2 (0.17)		
**Age group**					
15 to 20	370 (35.7))	83 (8.7)	262 (22.3)		
21 to 29	523 (50.5)	534 (55.8)	683 (58.1)		
30 to 39	137 (13.2)	287 (30.0)	249 (21.2)		
40 +	6 (0.6)	54 (5.6)	16 (1.4)		
**Education**					
Less than primary	153 (14.8)	107 (11.2)	62 (5.3)		
Primary completion	241 (23.3)	479 (50.0)	635 (54.0)		
Secondary	587 (56.7)	277 (28.9)	348 (29.6)		
Tertiary	55 (5.3)	95 (9.9)	132 (11.2)		
**Number of times pregnant**					
One	410 (39.6)	348 (36.4)	396 (33.7)		
Two	304 (29.3)	276 (28.8)	336 (28.6)		
Three	167 (16.1)	151 (15.8)	203 (17.3)		
Four or more	156 (15.1)	182 (19.0)	240 (20.4)		
**Marital status**					
Never married	0 (0.0)	10 (1.0)	136 (11.6)		
Ever married	1036 (100.0)	947 (99.0)	1001 (85.1)		

Among postnatal care clients, mean age was highest in Cambodia (26.8 years) and lowest in Bangladesh (23.8 years). Higher educational attainment (completion of secondary school or more) was greatest among postnatal clients in eSwatini (73.9%) and lowest among participants in the Kenya Integra study (19.1%). Postnatal clients in the Kenya Integra study were most likely to be primiparous (29.9%).

Among antenatal clients, mean age was slightly lower in Bangladesh (23.5) relative to Cambodia (27.8 years) and Kenya studies (25.2 years). On average, a higher proportion of antenatal clients in Bangladesh completed secondary school or more (62.0%) relative to those in Cambodia (38.8%) and Kenya (40.8%). Antenatal clients were most likely to be pregnant for the first time in Bangladesh (39.6%).

### Indicator validity across studies

**Fig. 2:**
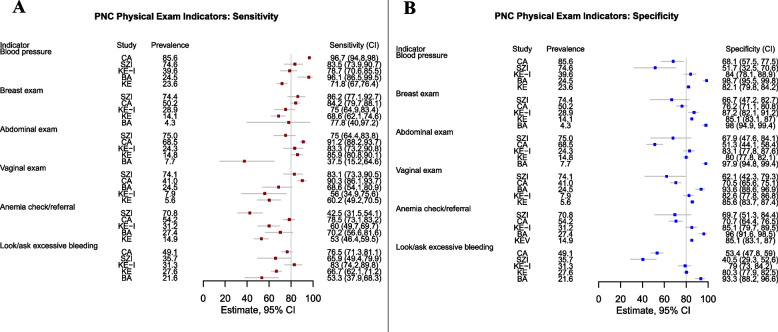
Postnatal Care (PNC) Indicator Sensitivity (Panel **A**) and Specificity (Panel **B**) by Country of Study, Sorted by Indicator Prevalence. Study abbreviations refer to Cambodia Voucher study (CA), Bangladesh Voucher study (BA), Kenya Voucher study (KE), Kenya Integra Study. (KE-I) and eSwatini Integra Study (SZI). Indicators are defined in Additional File 2. Grey horizontal lines represent 95% confidence intervals about the estimates. As a benchmark for indicator quality, 80% sensitivity and specificity is shown as a vertical grey line

**Fig. 3:**
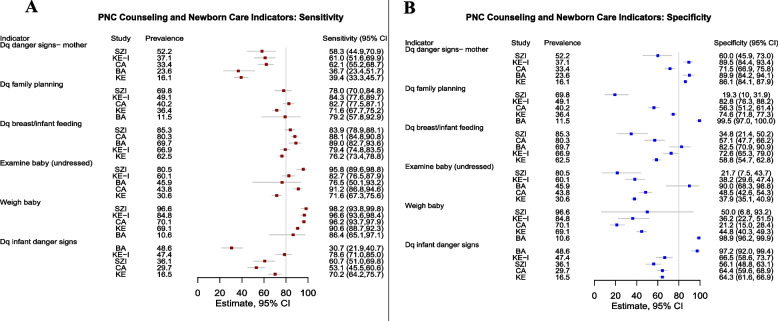
Postnatal Care (PNC) Counseling Indicator Sensitivity (Panel **A**) and Specificity (Panel **B**) by Country of Study, Sorted by Indicator Prevalence. Study abbreviations refer to Cambodia Voucher study (CA), Bangladesh Voucher study (BA), Kenya Voucher study (KE), Kenya. Integra Study (KE-I) and eSwatini Integra Study (SZI). Indicators are defined in Additional File 2. Dq abbreviation for “discussion of”. Grey horizontal lines represent 95% confidence intervals about the estimates. As a benchmark for indicator quality, 80% sensitivity and specificity is shown as a vertical grey line

Figures 2 and 3 display PNC indicator sensitivity and specificity across included studies. In general, indicators of PNC had higher sensitivity than specificity. With few noted exceptions, estimates of sensitivity and specificity demonstrated wide variability by study. One of twelve PNC indicators demonstrated a sensitivity of greater than 80% in all five studies—whether the infant was weighed. An additional six PNC indicators had a sensitivity of approximately 80% or higher in three of five studies – blood pressure check, breast exam, abdominal exam, discussion of family planning, infant physical exam (undressed), and discussion of breast/infant feeding. In contrast, no PNC indicator achieved a specificity of 80% or higher in all five studies. All PNC indicators which reflected aspects of the maternal physical exam also achieved a specificity of approximately 80% or more in three of five studies – blood pressure check, breast exam, abdominal exam, vaginal exam, anemia check/referral and whether the provider asked or checked for excessive bleeding. One counseling related indicator – whether dangers signs for the mother were discussed – also achieved a specificity of 80% or more in three of five studies. Few indicators of newborn PNC achieved a specificity of 80% or higher in any one study. No PNC indicator achieved both sensitivity and specificity greater than 80% in more than one study, underscoring considerable heterogeneity in validity results across settings.

ANC indicators performed similarly to PNC with generally high sensitivity across the three settings and variable specificity (Additional File 3). Three indicators of the maternal physical health checks during an ANC consultation had a sensitivity greater than 80% in all three studies: weight taken, blood pressure check and abdominal exam. While no ANC indicator had a specificity of greater than 80% in all three settings, two ANC indicators – urine screen and fetal heart rate monitoring – had a specificity of at least 80% in two or more settings. No ANC indicator had both sensitivity and specificity of 80% or higher in more than one study.

### Respondent characteristics: maternal age, education, and prior parity on PNC reporting accuracy

**Fig. 4:**
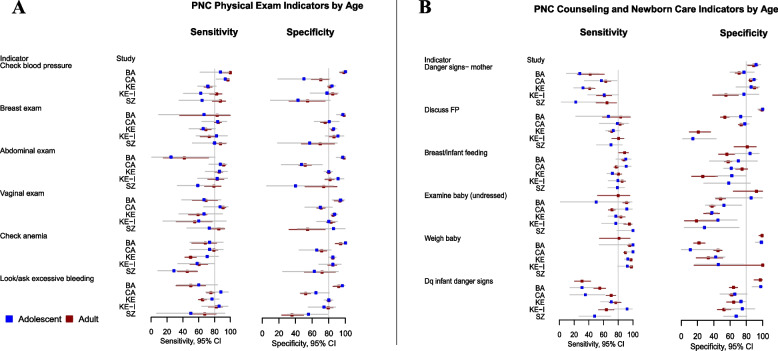
Postnatal Care (PNC) Indicator Sensitivity (Panel **A**) and Specificity (Panel **B**) by Country of Study, Stratified by Respondent Age Group. Study abbreviations refer to Cambodia Voucher study (CA), Bangladesh Voucher study (BA), Kenya Voucher study (KE), Kenya Integra. Study (KE-I) and eSwatini Integra Study (SZI). Age group: adolescent (ages 15 to 20 years), adult (ages >20 years). Grey horizontal lines represent 95% confidence intervals about the estimates, overlapping confidence intervals implies no statistical difference in level of the predictor. As a benchmark for indicator quality, 80% sensitivity and specificity is shown as a vertical grey line

Age-stratified results showed overlap in the 95% CI for sensitivity and specificity between adolescent and adult strata for all PNC indicators across studies (Fig. 4). Across individual studies, there was no clear pattern indicating that adolescent-reported sensitivity and specificity was better or worse than adult reporting. Wide confidence intervals in individual study estimates obscured any significant differences between age groups.

Estimates of sensitivity and specificity across postnatal care interventions (Table 3) stratified by adolescent and adult group also revealed substantial overlap in the 95% CI for all indicators, suggesting no differences by age. Similarly, no systematic differences between stratified bivariate models were observed for the predictors of education or prior parity for any postnatal care indicator examined (Tables 4 and 5).

**Table 3:** Bivariate random effects model: Self-reported PNC indicator accuracy by adolescent vs. adult age group

Indicator	Adolescent	Adult	Significant difference?
	Estimate (95% CI)	Estimate (95% CI)	
**Blood pressure check**			
Sensitivity	76.2 (60.8, 86.8)	90.2 (74.9, 96.6)	NA
Specificity	75.8 (49.3, 91.0)	82.2 (61.6, 93.0)	NA
**Breast exam**			
Sensitivity	74.9 (62.9, 0.840)	74.9 (59.8, 85.7)	NA
Specificity	85.9 (73.3, 93.2)	83.6 (72.4, 90.8)	N
**Abdominal exam**			
Sensitivity	74.7 (53.6, 88.3)	80.9 (65.1, 90.6)	N
Specificity	80.1 (52.0, 93.7)	84.1 (59.5, 95.0)	N
**Vaginal exam**			
Sensitivity	73.4 (59.4, 83.9)	75.6 (58.2, 87.4)	NA
Specificity	84.0 (74.0, 90.6)	80.1 (72.4, 86.1)	N
**Anemia check/referral**			
Sensitivity	62.3 (46.9, 75.5)	61.3 (47.7, 73.4)	NA
Specificity	80.1 (64.9, 89.7)	83.1 (73.3, 89.8)	NA
**Check/ask about excessive bleeding**			
Sensitivity	77.9 (66.6, 86.2)	69.6 (59.5, 78.2)	N
Specificity	77.3 (59.3, 88.9)	71.8 (47.8, 87.6)	NA
**Discuss danger signs for mother**			
Sensitivity	42.1 (28.0, 57.6)	54.7 (43.8, 65.2)	NA
Specificity	86.1 (79.3, 90.9)	80.5 (66.9, 89.5)	N
**Discuss family planning**			
Sensitivity	70.6 (62.7, 77.4)	77.9 (70.6, 83.8)	N
Specificity	68.2 (22.6, 96.1)	73.6 (20.5, 96.8)	NA
**Discuss breast/infant feeding**			
Sensitivity	81.9 (72.6, 88.5)	83.7 (78.8, 87.6)	N
Specificity	68.7 (58.5, 77.4)	60.2 (41.2, 76.6)	NA
**Examine baby (undressed)**			
Sensitivity	80.4 (71.7, 86.9)	85.8 (75.6, 92.1)	N
Specificity	43.1 (33.8, 52.9)	43.1 (38.3, 48.1)	N
**Weigh baby**			
Sensitivity	92.3 (87.4, 95.4)	93.2 (85.9, 96.8)	Y
Specificity	58.3 (13.6, 92.6)	61.8 (18.1, 92.2)	NA
**Give information on baby sickness signs**			
Sensitivity	57.2 (31.6, 79.4)	59.8 (43.2, 74.5)	N
Specificity	72.1 (67.0, 76.8)	71.5 (48.1, 87.2)	NA

Comparison of estimates in grey have been suppressed due to low precision (15 percentage points or more). Adolescent age group: 15-20 years; Adult age group: >20 years

**Table 4:** Bivariate random effects model: Self-reported PNC indicator accuracy by education

Indicator	Less than primary school completion	Primary completion or higher	Significant difference?
	Estimate (95% CI)	Estimate (95% CI)	
**Blood pressure check**			
Sensitivity	88.8 (75.9, 95.2)	85.3 (63.2, 95.2)	N
Specificity	83.0 (67.3, 92.1)	83.2 (56.6, 94.9)	N
**Breast exam**			
Sensitivity	76.1 (54.4, 84.9)	72.9 (56.4, 84.8)	N
Specificity	83.3 (73.1, 90.2)	86.6 (71.9, 94.3)	N
**Abdominal exam**			
Sensitivity	82.2 (65.9, 91.7)	76.9 (59.0, 88.5)	N
Specificity	82.7 (58.2, 94.3)	83.6 (59.6, 94.6)	N
**Vaginal exam**			
Sensitivity	71.9 (53.8, 84.9)	79.0 (62.3, 89.6)	N
Specificity	80.3 (69.6, 87.9)	83.0 (72.5, 90.1)	N
**Anemia check/referral**			
Sensitivity	64.5 (55.6, 72.6)	59.6 (41.6, 75.3)	NA
Specificity	81.8 (73.2, 88.1)	84.1 (73.7, 90.9)	N
**Check/ask excessive bleeding**			
Sensitivity	71.8 (58.7, 82.0)	69.7 (63.4, 75.3)	N
Specificity	73.2 (54.6, 86.1)	74.5 (46.8, 90.7)	NA
**Discuss danger signs for mother**			
Sensitivity	53.0 (40.6, 65.0)	48.3 (36.2, 60.7)	N
Specificity	85.0 (75.5, 91.3)	79.8 (67.7, 88.2)	N
**Discuss family planning**			
Sensitivity	80.1 (72.5, 86.0)	75.4 (68.6, 81.1)	N
Specificity	73.8 (30.8, 94.7)	74.5 (33.7, 94.4)	NA
**Discuss breast/infant feeding**			
Sensitivity	83.3 (77.5, 87.9)	83.3 (74.5, 89.5)	N
Specificity	68.1 (59.6, 75.5)	55.5 (34.2, 75.0)	NA
**Examine baby (undressed)**			
Sensitivity	85.0 (73.4, 92.1)	82.1 (65.9, 91.5)	N
Specificity	41.1 (34.9, 47.6)	44.6 (28.4, 62.0)	NA
**Weigh baby**			
Sensitivity	93.9 (87.7, 97.1)	92.2 (84.6, 96.3)	N
Specificity	50.3 (14.6, 85.7)	62.7 (17.2, 93.2)	NA
**Give information on baby sickness signs**			
Sensitivity	57.1 (35.0, 76.6)	56.2 (43.4, 68.3)	NA
Specificity	65.9 (63.0, 68.6)	73.3 (50.8, 87.9)	N

Comparison of estimates in grey have been suppressed due to low precision (15 percentage points or more)

**Table 5:** Bivariate random effects model: self-reported PNC indicator accuracy by parity

Indicator	First birth	One or more prior births	Significant difference?
	Estimate (95% CI)	Estimate (95% CI)	
**Blood pressure check**			
Sensitivity	86.4 (67.3, 95.1)	85.9 (72.8, 93.3)	NA
Specificity	75.8 (67.0, 82.9)	77.9 (70.2, 84.1)	N
**Breast exam**			
Sensitivity	79.3 (70.4, 86.0)	77.1 (66.9, 84.9)	N
Specificity	80.5 (75.1, 85.0)	82.8 (76.8, 87.5)	N
**Abdominal exam**			
Sensitivity	85.0 (78.8, 89.6)	84.0 (75.7, 89.9)	N
Specificity	76.2 (60.7, 86.9)	79.0 (65.4, 88.2)	NA
**Vaginal exam**			
Sensitivity	71.4 (47.4, 87.3)	73.6 (56.9, 85.4)	NA
Specificity	78.1 (68.7, 85.3)	81.3 (74.7, 86.5)	N
**Anemia check/referral**			
Sensitivity	58.3 (45.2, 70.4)	61.9 (47.5, 74.5)	N
Specificity	79.6 (71.9, 85.9)	83.1 (77.6, 87.5)	N
**Check/ask excessive bleeding**			
Sensitivity	71.9 (65.9, 77.3)	72.0 (63.0, 79.5)	N
Specificity	65.6 (45.7, 88.2)	67.7 (51.0, 80.9)	NA
**Discuss danger signs for mother**			
Sensitivity	45.2 (32.3, 58.9)	55.8 (44.1, 67.0)	N
Specificity	79.9 (64.8, 89.5)	80.0 (69.8, 87.4)	N
**Discuss breast/infant feeding**			
Sensitivity	81.4 (73.5, 87.3)	81.3 (75.3, 86.1)	N
Specificity	64.3 (58.4, 69.8)	62.3 (40.5, 80.0)	NA
**Examine baby (undressed)**			
Sensitivity	86.3 (76.5, 92.4)	83.5 (71.9, 90.9)	N
Specificity	48.1 (40.5, 55.8)	40.2 (35.9, 44.6)	N
**Weigh baby**			
Sensitivity	89.6 (79.6, 95.0)	93.6 (86.2, 97.1)	N
Specificity	54.0 (25.4, 80.3)	36.8 (21.5, 55.4)	NA
**Give information on baby sickness signs**			
Sensitivity	60.7 (46.8, 73.1)	66.8 (54.6, 77.1)	N
Specificity	69.6 (62.3, 75.9)	60.7 (52.7, 68.2)	N

Comparison of estimates in grey have been suppressed due to low precision (15 percentage points or more). Whether family planning was discussed was not analyzed due to fewer than five studies with sufficient sample size

The same general patterns were observed in univariate fixed effects estimates obtained for ANC indicators by age, education, and parity (Additional files 4 and 5). Although there were some exceptions, for most indicators there were either no differences by subgroup or comparison was not possible due to low precision.

### Facility quality

Differences in the accuracy of PNC indicators were inconsistent by facility quality (whether respondents attended a voucher intervention facility or comparable control facility) (Additional file 6). Of eight indicators with reasonable precision for comparison, two indicators differed by facility quality level but in mixed directions. The odds of correct reporting on whether the infant was examined (undressed) was greater among respondents who visited non-voucher facilities (proxy for lower facility quality), while whether information on infant danger signs was discussed was more likely to reported accurately among mothers who attended voucher intervention relative to control facilities (proxy for higher facility quality).

### Intervention coverage

Visual inspection of paired forest plots of sensitivity and specificity for PNC indicators sorted by intervention coverage (Figs. 2 and 3) illustrate that, for most indicators, there is a trend of decreasing specificity (more false positive reporting) with higher levels of intervention coverage across studies. In the forest plots, there is some evidence that indicator sensitivity improves with increasing prevalence (most apparent for indicators of the maternal physical exam), however, this pattern is less strong.

Results of the likelihood ratio test which compared model fit for a bivariate random effects model that incorporated intervention coverage as a study-level covariate relative to an intercept-only model confirmed that intervention coverage significantly explained heterogeneity in reporting accuracy between studies for the majority (9 of 12) indicators (Table 6). Separate tests that examined the influence of intervention coverage on indicator reporting accuracy demonstrate that indicator specificity decreased with higher intervention coverage levels for the majority (8) of indicators, implying greater false positive reporting. Results also show that increased sensitivity was also positively associated with intervention coverage for half (6) of the indicators, implying low false negative reporting. The relationship between intervention coverage and indicator sensitivity and specificity was variable among ANC indicators (Additional File 6), with only three studies per indicator.

**Table 6:** Influence of intervention coverage on self-reported PNC indicator accuracy^1^

	**Bivariate random effects model adjusted by intervention coverage**		**Likelihood Ratio Test**^**2**^		**Predicted Sensitivity (SE), False Positive Rate (FPR) and Specificity (SP) at Varying Intervention Coverage Levels**^3^			
	**Log odds (95% CI)**	***p*-value**	${\varvec{\chi}}$ ^**2**^	***p*-value**	**Measure**	**25%**	**50%**	**75%**
**Blood pressure check**								
SE Intercept	1.40 (-0.30, 3.11)	0.107	26.48	< 0.001	SE	85.2	89.1	92.1
SE.Coverage	0.01 (-0.02, 0.04)	0.371			FPR	8.6	18.4	31.2
FPR Intercept	-3.24 (-4.96, -1.52)	< 0.001			SP	91.4	81.6	68.8
FPR.Coverage	0.04 (0.00, 0.07)	0.025						
**Breast exam**								
SE Intercept	0.69 (0.30, 1.08)	0.001	12.98	0.002	SE	75.7	83.0	88.5
SE.Coverage	0.02 (0.01, 0.03)	< 0.001			FPR	11.8	22.5	35.1
FPR Intercept	-2.79 (-3.54, -2.03)	< 0.001			SP	88.2	77.5	64.9
FPR.Coverage	0.03 (0.01, 0.05)	0.001						
**Vaginal exam**								
SE Intercept	0.44 (-0.29, 1.16)	0.241	18.6	< 0.001	SE	72.8	82.3	88.9
SE.Coverage	0.02 (0.00, 0.04)	0.018			FPR	18.9	25.3	31.4
FPR Intercept	-1.83 (-2.18, -1.49)	< 0.001			SP	81.1	74.7	68.6
FPR.Coverage	0.02 (0.00, 0.03)	0.005						
**Abdominal exam**								
SE Intercept	0.67 (-0.64, 1.98)	0.314	10.44	0.005	SE	74.5	81.3	86.7
SE.Coverage	0.02 (-0.01, 0.04)	0.246			FPR	13.2	25.3	39.1
FPR Intercept	-2.68 (-3.80, -1.57)	< 0.001			SP	86.8	74.7	60.9
FPR.Coverage	0.03 (0.01, 0.06)	0.008						
**Anemia check/referral**								
SE Intercept	0.58 (-0.52, 1.67)	0.300	3.53	0.171	SE	62.9	61.8	60.6
SE.Coverage	0.00 (-0.03, 0.02)	0.843			FPR	12.7	22.2	32.8
FPR Intercept	-2.61 (-3.58, -1.63)	< 0.001			SP	87.3	77.8	67.2
FPR.Coverage	0.03 (0.00, 0.05)	0.023						
**Check/ask about excessive bleeding**								
SE Intercept	0.08 (-0.44, 0.60)	0.766	6.00	0.050	SE	65.8	77.4	85.9
SE.Coverage	0.02 (0.01, 0.04)	0.002			FPR	15.1	60.4	89.5
FPR Intercept	-3.88 (-5.87, -1.88)	< 0.001			SP	84.9	39.6	10.5
FPR.Coverage	0.09 (0.03, 0.14)	0.003						
**Discuss danger signs for mother**								
SE Intercept	-0.85 (-1.50, -0.20)	0.011	6.10	0.047	SE	47.6	65.8	80.3
SE.Coverage	0.03 (0.01, 0.05)	0.003			FPR	14.7	30.9	48.8
FPR Intercept	-2.71 (-3.93, -1.48)	< 0.001			SP	85.3	69.1	51.2
FPR.Coverage	0.04 (0.00, 0.07)	0.039						
**Discuss family planning**								
SE Intercept	-2.73 (-4.39, -1.07)	0.001	1.827	0.401	SE	25.8	65.0	90.8
SE.Coverage	0.07 (0.03, 0.10)	< 0.001			FPR	41.8	59.7	72.6
FPR Intercept	-1.06 (-4.45, 2.34)	0.542			SP	58.2	40.3	27.4
FPR.Coverage	0.03 (-0.04, 0.10)	0.43						
**Discuss breast/infant feeding**								
SE Intercept	-0.10 (-1.86, 1.67)	0.915	13.32	0.001	SE	62.3	75.1	84.6
SE.Coverage	0.02 (0.00, 0.05)	0.058			FPR	4.9	15.9	34.9
FPR Intercept	-4.26 (-8.57, 0.04)	0.052			SP	95.1	84.1	65.1
FPR.Coverage	0.05 (-0.01, 0.11)	0.085						
**Examine baby (undressed)**								
SE Intercept	0.40 (-1.02, 1.82)	0.581	11.31	0.004	SE	75.0	85.8	92.4
SE.Coverage	0.03 (0.00, 0.06)	0.051			FPR	57.9	59.7	61.2
FPR Intercept	0.25 (-0.48, 0.97)	0.509			SP	42.1	40.3	38.8
FPR.Coverage	0.00 (-0.01, 0.02)	0.752						
**Weigh baby**								
SE Intercept	1.29 (-0.23, 2.81)	0.096	5.6	0.061	SE	86.3	91.6	95.0
SE.Coverage	0.02 (0.00, 0.04)	0.038			FPR	5.4	22.5	51.6
FPR Intercept	-4.49 (-6.72, -2.25)	< 0.001			SP	94.6	77.5	48.4
FPR.Coverage	0.07 (0.03, 0.10)	< 0.001						
**Give information on baby sickness signs**								
SE Intercept	1.02 (-0.85, 2.89)	0.283	9.33	0.009	SE	79.8	79.0	78.1
SE.Coverage	-0.02 (-0.07, 0.03)	0.479			FPR	4.7	15.0	32.9
FPR Intercept	0.60 (-1.87, 3.06)	0.635			SP	95.3	85.0	67.1
FPR.Coverage	-0.05 (-0.11, 0.02)	0.180						

^1^ For all indicators where at least 5 studies, *SE* Sensitivity, *FPR* False positive rate (1-specificity). A positive effect coefficient for SE.Prevalence implies a positive relationship between intervention coverage level and sensitivity. A positive effect coefficient between intervention coverage level and the false positive rate implies higher false positive reporting (decreased specificity) with higher intervention coverage level

^2^ Likelihood ratio test comparing model fit of adjusted versus intercept-only model at 2 degrees of freedom. Significant result for LLRT indicates preferred fit for adjusted model

^3^ Effect of changing intervention coverage on SE (or Specificity using 1-FPR) is obtained using following example formula: plogit(SE.Intercept + Coverage Level* SE.Coverage) where plogit = (exp(log odds))/(1 + (exp(log odds)) *** < 0.0001, ** < 0.001, * < 0.05

## Discussion

We assessed heterogeneity in self-reported antenatal and postnatal care by respondent and facility characteristics using data from five studies across Sub-Saharan Africa and Southeast Asia. Results show that no indicator of antenatal care nor postnatal care achieved a combined high sensitivity and specificity (80% or higher) in more than one study, underscoring variability in validity estimates across settings. We also did not find strong evidence that accuracy in self-reported ANC or PNC care systematically varied by maternal characteristics, such as adolescent vs. adult age, education, parity, or by facility quality. Higher intervention coverage level, however, was associated with reduced specificity (higher false positive reporting) and somewhat improved sensitivity (lower false negative reporting) for most indicators.

That validity did not systematically vary by respondent characteristics or facility quality is perhaps a surprising, although reassuring result in terms of approaches to data collection and indicator construction. Our finding is largely consistent with prior studies of respondent characteristics on reporting accuracy for received maternal health services which have found that associations vary by both indicator and respondent attribute [30, 31, 47]. In addition, no consistent evidence related to facility quality (voucher intervention or control facility) was observed across indicators. This finding aligns with a study which assessed how the accuracy of women’s perceptions of facility quality predicted her choice of where to receive care in informal settlements of Nairobi, Kenya. The study found substantial evidence of ‘information asymmetry’ – that a high proportion of women (two in five) were unable to discern which facilities offered the highest technical quality of care prior to using the facility’s services [48]. It may be that inaccurate perceptions of facility quality explain, in part, why facility quality was inconsistently related to reporting accuracy. It is also possible that women value different aspects of care, including the patient care experience, than those typically emphasized in monitoring efforts [49]. Our measure of facility quality may have been an incomplete proxy for how women perceive quality care.

The finding that higher study intervention coverage (i.e., prevalence) is associated with reduced specificity and somewhat improved sensitivity is also in accordance with prior findings and has important implications for efforts to monitor maternal and newborn quality of care. While sensitivity and specificity are independent of prevalence in their mathematical calculation, several studies and reviews have suggested an association [50]. A study by Carter and colleagues, which assessed the reliability of maternal recall of delivery and immediate newborn care indicators in Nepal, for example, also documented an inverse association between indicator specificity and higher intervention coverage [47]. This pattern may be the result of reporting biases in the classification of the reference standard (i.e., the observer report) and/or in women’s self-reports (the ‘test’) [50]. For example, it is possible that in settings where an intervention is commonplace, respondents are more likely to anticipate that it will occur and in turn respond affirmatively. This type of reporting bias would lead to higher false positive reporting (lower specificity), implying monitoring efforts would overestimate coverage in high coverage settings. A high expectation of care could also imply few false negative reports (high sensitivity), which was observed for about half of the indicators in our analysis. In high coverage settings women who did receive the intervention were unlikely to be undercounted. However, in low coverage settings, underestimation (low sensitivity) may be an issue. For monitoring progress in the quality of maternal and newborn care, the reduced specificity in high prevalence settings and lower sensitivity in low prevalence settings is of public health importance. Although descriptive only, our results suggest that monitoring efforts should consider the context of care when interpreting national estimates and time trends in intervention coverage, as mismeasurement may occur in both directions dependent on setting.

A strength of this study is that we were able to synthesize patterns in reporting accuracy across several studies which used the exact or very similar question wording and recall time by interviewing women at facility discharge for a routine antenatal or postnatal care visit. This addresses the limitations of prior studies on this subject which have not been able to discern patterns across settings and have smaller sample sizes for subgroup analysis. The ability to examine validation results across settings descriptively and with statistical assessment lends robustness to our main findings. However, several important limitations remain. Primarily, few studies have examined the accuracy of maternal reports of antenatal and postnatal care using comparable indicators and it is possible that a relevant study was missed. Further research to examine variability in indicator accuracy across settings is warranted. The few number of studies assessed contributed to low precision in our analysis, particularly for ANC indicators which were only assessed in three studies and used fixed, rather than random effects models. Results from the fixed effects models should be considered exploratory as variability by study, correlation between sensitivity and specificity, and heterogeneity attributed to threshold differences across studies are not accounted for. For example, observer training for what constituted an intervention having taken place may have varied across studies. Further, it was possible to incorporate study aggregate variables (i.e., intervention coverage) only, rather than within-study covariates (e.g., respondent age, education parity) [41]. To assess variability by respondent individual characteristics we used stratification, which reduces precision. For example, the sub-sample of adolescents across studies was relatively small, despite increasing the age category to include respondents aged 20 years. Finally, given data availability, it was not possible to examine facility type (e.g., public sector or not, tier of facility) across studies. This is a topic for future research. We hypothesize that intervention coverage within facility type may, at least in part, contribute to observed differences in validity.

Despite noted limitations, the finding that reporting accuracy does not consistently vary by respondent or facility characteristics is reassuring news for efforts to monitor the quality of maternal and newborn care. Evidence of consistently lower reporting accuracy by respondent characteristics such as adolescent age could, for example, suggest that self-reported data may be insufficient to inform country-level interventions, policies and resource allocation for a group at high risk of adverse maternal or infant health outcomes ([25, 26], and this was not the case. However, study findings do suggest that caution is warranted when interpreting results, obtained by participant self-report, of interventions to improve quality of maternal and newborn care in very low, or alternatively very high, prevalence settings as false negative and false positive reporting may be more likely in either setting. National monitoring efforts should consider the context of care in the interpretation of country estimates of the coverage of self-reported quality of care and triangulate with other available data sources such as facility registries. Further research to validate indicators in additional study settings and which models the extent different intervention coverage levels affect the ability to detect changes in coverage between countries and over time is warranted. With sufficient confidence in such models, adjustment factors could be applied to coverage estimates in global monitoring efforts to account for bias attributed to differences in intervention prevalence. At the very least, caution is warranted in the interpretation of coverage estimates from very high or low prevalence settings.

## Conclusions

Results from this study provide no evidence to suggest that self-reported receipt of maternal and newborn health interventions are consistently influenced by respondent characteristics including adolescent vs. adult age group, education, parity or facility quality. Rather, this analysis suggests that accuracy differences across studies is, at least in part, explained by differences in the prevalence of the intervention across settings. This study suggests that high-intervention coverage settings may contribute to higher false positive reporting (poorer specificity) among women who receive PNC care at health facilities and undercount intervention coverage (lower sensitivity) in low prevalence settings. Caution may be warranted when interpreting population-based household survey estimates of quality, or change in quality over time, in very high or very low prevalence settings.

## Supplementary Information

**Additional file 1.** Antenatal care (ANC) indicator construction.

**Additional file 2.** Postnatal care (PNC) indicator construction.

**Additional file 3.** Antenatal Care Indicator Sensitivity (Panel A) and Specificity (Panel B) by Country of Study, Sorted by Indicator Prevalence. Bangladesh (BA), Cambodia (CA) and Kenya (KE).

**Additional file 4.** Antenatal Care Indicator Sensitivity and Specificity by Country of Study and Age Group (Adolescent vs. Adult).

**Additional file 5.** Univariate fixed effects model: self-reported ANC indicator accuracy by respondent and facility characteristics.

**Additional file 6.** Univariate fixed effects models: Self-reported PNC indicator accuracy by facility quality (non-voucher vs. voucher intervention facility).

## Data Availability

The datasets analyzed during the current study are available from the corresponding author on reasonable request.

## Abbreviations

ANC: Antenatal care
DHS: Demographic health survey
MICS: Multiple indicator cluster survey
PNC: Postnatal care
AUC: Area under the receiver operating curve
IF: Inflation factor
