# Supplementary material for: Exploring the accuracy of self-reported maternal and newborn care in select studies from low and middle-income country settings: do respondent and facility characteristics affect measurement?
Source: BMC Pregnancy Childbirth. 2023 Jun 16;23:448. doi: 10.1186/s12884-023-05755-7 (PMC10273708; doi:10.1186/s12884-023-05755-7)
Supplement: Supplementary file 1 — Additional file 1. Antenatal care (ANC) indicator construction. [file 12884_2023_5755_MOESM1_ESM.docx]

Additional File 1. Antenatal care (ANC) indicator construction.

| **Indicator** |  | **Bangladesh** | **Cambodia** | **Kenya** |
| --- | --- | --- | --- | --- |
| Measure weight | Client | During your visit today, did the staff…take your weight? | During your visit today, did the staff…take your weight? | During your visit today, did the staff…take your weight? |
|  | Observer | Take client’s weight | Take client’s weight | Take client’s weight |
| Blood pressure check | Client | Check your blood pressure | Check your blood pressure | Check your blood pressure |
|  | Observer | Take client’s blood pressure | Take client’s blood pressure | Take client’s blood pressure |
| Abdominal exam | Client | Examine lower abdomen for fetal presentation | Perform abdominal examination | Perform abdominal examination |
|  | Observer | Palpate abdomen for fetal presentation | Palpate the client's abdomen for fundal height OR Palpate abdomen for fetal presentation OR Palpate abdomen for engagement of presenting parts | Palpate the client's abdomen for fundal height OR Palpate abdomen for fetal presentation OR Palpate abdomen for engagement of presenting parts |
| Check anemia (pallor or refer for HB test) | Client | [Baseline] Check for anemia [Follow-up] Check conjunctiva (eyelids) OR Blood test for hemoglobin | Check for anemia | Check for anemia |
|  | Observer | Check conjunctiva or refer/test blood | Observer/test for anemia OR Check the woman’s conjunctiva (eyelids) | Observe/test for anemia OR Check the woman’s conjunctiva (eyelids) |
| Check fetal heart rate | Client | Check fetal pulse rate | Listen to the baby’s heartbeat | Listen to the baby’s heartbeat |
|  | Observer | Listen to fetal heart rate | Listen to fetal heart rate | Listen to fetal heart rate |
| Urine screen | Client | [Baseline]: Had lab test (urine); [Follow-up] Lab test (urine) for protein OR Lab test (urine) for glucose OR Lab test (urine) for albumin OR Lab test (urine) for others | Check urine for protein and sugar | Check urine for protein and sugar |
|  | Observer | Urine test or refer client for urine bacterium | Urine test or refer client for urine bacterium | Urine test or refer client for urine bacterium |
|  | Observer | Sacmo/medical assistant |  |  |
