# Supplementary material for: Exploring the accuracy of self-reported maternal and newborn care in select studies from low and middle-income country settings: do respondent and facility characteristics affect measurement?
Source: BMC Pregnancy Childbirth. 2023 Jun 16;23:448. doi: 10.1186/s12884-023-05755-7 (PMC10273708; doi:10.1186/s12884-023-05755-7)
Supplement: Supplementary file 2 — Additional file 2. Postnatal care (PNC) indicator construction. [file 12884_2023_5755_MOESM2_ESM.docx]

| Additional File 2: Postnatal care (PNC) indicator construction. | | | | | | |
| --- | --- | --- | --- | --- | --- | --- |
|  |  | Voucher Study | | | Integra Study | |
|  |  | Bangladesh | Cambodia | Kenya | eSwatini | Kenya |
| Blood pressure check | Client | Measure your blood pressure | Measure your blood pressure | Measure your blood pressure | Measure your blood pressure | Measure your blood pressure |
|  | Observer | Take client’s blood pressure | Take client’s blood pressure | Take client’s blood pressure | Take client’s blood pressure | Take client’s blood pressure |
| Breast exam | Client | Examine your breasts | Examine your breasts | Examine your breasts | Examine your breasts | Examine your breasts |
|  | Observer | Examined breasts and nipples | Examined breasts and nipples | Examined breasts and nipples | Examined breasts and nipples | Examined breasts and nipples |
| Abdominal exam | Client | *Examine height of uterus* | Examine your abdomen | Examine your abdomen | Examine your abdomen | Examine your abdomen |
|  | Observer | Lower abdominal examination for uterine involution | Palpate the client’s abdomen for uterine involution | Palpate the client’s abdomen for uterine involution | Palpate the client’s abdomen for uterine involution | Palpate the client’s abdomen for uterine involution |
| Vaginal exam | Client | *Did you have vaginal bleeding/perineal/episiotomy examination in PNC* | Examine the vagina | Examine the vagina | Examine your vagina | Examine your vagina |
|  | Observer | *Checked/asked about extent of vaginal bleeding/perineal tear/examined episiotomy/ checked vaginal discharge* | Pelvic examination /checked perineum | Pelvic examination /checked perineum | Pelvic examination / checked perineum | Pelvic examination / checked perineum |
| Check anemia (pallor or refer for HB test) | Client | *Did you have an anemia examination* | Check you for anemia | Check you for anemia | Check you for anemia | Check you for anemia |
|  | Observer | *Checked woman's conjunctiva for anemia* | Check for pallor (anemia) /Refer for anemia test (HB) | Check for pallor (anemia) /Refer for anemia test (HB) | Check for pallor (anemia) /Refer for anemia test (HB) | Check for pallor (anemia) /Refer for anemia test (HB) |
| Check/ask about excessive bleeding | Client | Did you have a vaginal bleeding examination | Ask if you had any abnormal bleeding | Ask if you had any abnormal bleeding | Ask if you had any abnormal bleeding | Ask if you had any abnormal bleeding |
|  | Observer | Checked/asked about extent of peri-vaginal bleeding [Or] *Checked if bleeding since birth* | Checked/asked about extent of peri-vaginal bleeding | Checked/asked about extent of peri-vaginal bleeding | Checked/asked about extent of peri-vaginal bleeding | Checked/asked about extent of peri-vaginal bleeding |
| Discuss danger signs for mother | Client | Inform on possible PNC related problems/ complications | Discuss with you signs of complications after birth | Discuss with you signs of complications after birth | Discuss with you danger signs after birth; *Discuss with you complications after birth* | Discuss with you danger signs after birth |
|  | Observer | Discussed any of following: foul smelling vaginal discharge, fever with or without chills, excessive vaginal bleeding, broken stitches (perineal), cracked nipples, painful engorged breasts | Discussed any of following: foul smelling vaginal discharge, fever with or without chills, excessive vaginal bleeding, broken stitches (perineal), cracked nipples, painful engorged breasts | Discussed any of following: foul smelling vaginal discharge, fever with or without chills, excessive vaginal bleeding, broken stitches (perineal), cracked nipples, painful engorged breasts | Discussed any of following [foul smelling discharge; fever w/ or w/o chills; excessive vaginal bleeding; broken scars (perineum/ cesarean; painful crack/bleeding nipples; painful engorged breasts) | Discussed any of following [foul smelling discharge; fever w/ or w/o chills; excessive vaginal bleeding; broken scars (perineum/ cesarean; painful crack/bleeding nipples; painful engorged breasts) |
| Discussed with you family planning | Client | *Did the service provider inform or advice on… child spacing or use of family planning after delivery [Or] Various family planning methods* | Discuss with you family planning | Discuss with you family planning | Discuss with you family planning / *give information about family planning* | Discuss with you family planning / *give information about family planning* |
|  | Observer | Discuss healthy timing and birth spacing or use family planning after delivery [Or] *Discuss health benefits for mother and baby when birth spacing* [Or] Discuss various family planning methods | Discuss healthy timing and spacing of pregnancies /family planning [Or] Discuss or advise on the various family planning methods | Discuss healthy timing and spacing of pregnancies /family planning [Or] Discuss or advise on the various family planning methods | Discuss healthy timing and spacing of pregnancies /family planning [Or] Discuss or advise on the various family planning methods | Discuss healthy timing and spacing of pregnancies /family planning [Or] Discuss or advise on the various family planning methods |
| Discuss breast / infant feeding | Client | *Counseled on breastfeeding for baby [Or] Discuss with you about breastfeeding* | Discuss breastfeeding/feeding for the baby | Discuss breastfeeding/feeding for the baby | Discuss breastfeeding/feeding for the baby | Discuss breastfeeding/feeding for the baby |
|  | Observer | Yes to any of following: Discussed infant feeding, Encouraged mother to discuss how she was managing with breastfeeding, Re-emphasized exclusive feeding, Emphasized NO to mixed feeding | Yes to any of following: Discussed infant feeding, Encouraged mother to discuss how she was managing with breastfeeding, Re-emphasized exclusive feeding, Emphasized NO to mixed feeding | Yes to any of following: Discussed infant feeding, Encouraged mother to discuss how she was managing with breastfeeding, Re-emphasized exclusive feeding, Emphasized NO to mixed feeding | Yes to any of following: Discussed infant feeding, Encouraged mother to discuss how she was managing with breastfeeding, Re-emphasized exclusive feeding, Emphasized NO to mixed feeding | Yes to any of following: Discussed infant feeding, Encouraged mother to discuss how she was managing with breastfeeding, Re-emphasized exclusive feeding, Emphasized NO to mixed feeding |
| Examine baby (undressed) | Client | *Physical examination of baby* | Examine the baby (physical check, unclothed) | Examine the baby (physical check, unclothed) | Examine the baby (physical check, unclothed) | Examine the baby (physical check, unclothed) |
|  | Observer | Examine the baby (undressed) | Examine the baby (undressed) | Examine the baby (undressed) | Examine the baby (undressed) | Examine the baby (undressed) |
| Weigh the baby | Client | Baby's weight measured | Baby's weight measured | Baby's weight measured | Baby's weight measured | Baby's weight measured |
|  | Observer | Did the provider weigh the baby | Did the provider weigh the baby | Did the provider weigh the baby | Did the provider weigh the baby | Did the provider weigh the baby |
| Gave information on baby's sickness signs | Client | Counseled on sickness signs for baby | Give you information on the baby’s sickness signs | Give you information on the baby’s sickness signs | Did the provider give you information on the baby's sickness signs; *Did any provider tell you about danger signs that you should look out for in the baby* | Did the provider give you information on the baby's sickness signs; *Did any provider tell you about danger signs that you should look out for in the baby* |
|  | Observer | Discussed any of following: difficulty in breathing; poor feeding; jaundice (yellow skin/eyes); bleeding from cord; redness, swelling and/or pus around cord; baby feels hot or cold; abnormal crying; abdominal distension/vomiting; septic spots/boils on body; lethargy; convulsions | Discuss infant danger signs (any one of following): feeding difficulties- not sucking or sucking poorly, breathing difficulties, body feels hot or too cold, jaundice - yellow skin or eyes. | Discuss infant danger signs (any one of following): feeding difficulties- not sucking or sucking poorly, breathing difficulties, body feels hot or too cold, jaundice - yellow skin or eyes. | Discuss infant danger signs (any one of following): feeding difficulties- not sucking or sucking poorly, breathing difficulties, body feels hot or too cold, jaundice - yellow skin or eyes. | Discuss infant danger signs (any one of following): feeding difficulties- not sucking or sucking poorly, breathing difficulties, body feels hot or too cold, jaundice - yellow skin or eyes. |

*Italicized text indicates wording differences across studies.
