# Supplementary material for: Exploring the accuracy of self-reported maternal and newborn care in select studies from low and middle-income country settings: do respondent and facility characteristics affect measurement?
Source: BMC Pregnancy Childbirth. 2023 Jun 16;23:448. doi: 10.1186/s12884-023-05755-7 (PMC10273708; doi:10.1186/s12884-023-05755-7)
Supplement: Supplementary file 3 — Additional file 3. Antenatal Care Indicator Sensitivity (Panel A) and Specificity (Panel B) by Country of Study, Sorted by Indicator Prevalence. Bangladesh (BA), Cambodia (CA) and Kenya (KE). [file 12884_2023_5755_MOESM3_ESM.docx]

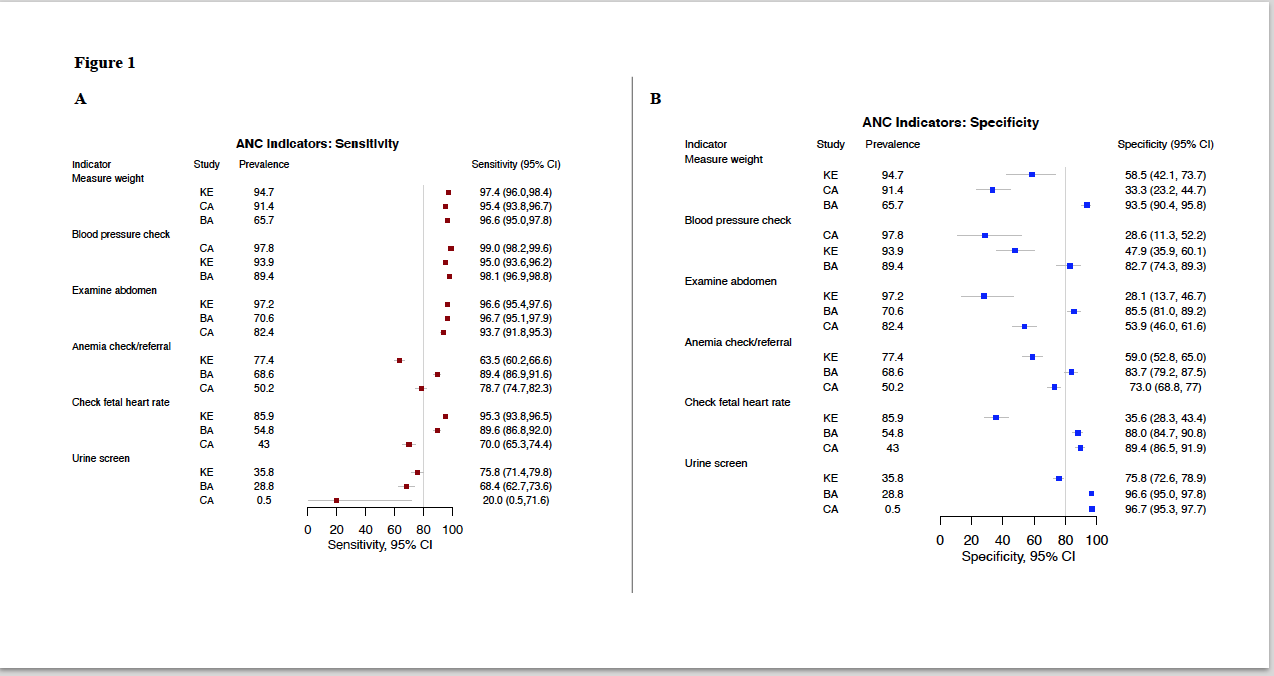


Additional File 3. Antenatal Care Indicator Sensitivity (Panel A) and Specificity (Panel B) by Country of Study, Sorted by Indicator Prevalence. Bangladesh (BA), Cambodia (CA) and Kenya (KE). Grey horizontal lines represent 95% confidence intervals about the estimates, overlapping confidence intervals implies no statistical difference in level of the predictor. As a benchmark for indicator quality, 80% sensitivity and specificity is shown as a vertical grey line
