# Supplementary material for: Exploring the accuracy of self-reported maternal and newborn care in select studies from low and middle-income country settings: do respondent and facility characteristics affect measurement?
Source: BMC Pregnancy Childbirth. 2023 Jun 16;23:448. doi: 10.1186/s12884-023-05755-7 (PMC10273708; doi:10.1186/s12884-023-05755-7)
Supplement: Supplementary file 4 — Additional file 4. Antenatal Care Indicator Sensitivity and Specificity by Country of Study and Age Group (Adolescent vs. Adult). [file 12884_2023_5755_MOESM4_ESM.docx]

**Additional File 4.**

Additional File 4. Antenatal Care Indicator Sensitivity and Specificity by Country of Study and Age Group. Country of study: Bangladesh (BA), Cambodia (CA) and Kenya (KE). Adolescent age group: ages 15 to 20 years, Adult age group - ages 21 to 52 years. Grey horizontal lines represent 95% confidence intervals about the estimates, overlapping confidence intervals implies no statistical difference in level of the predictor. As a benchmark for indicator quality, 80% sensitivity and specificity is shown as a vertical grey line.
