# Supplementary material for: Exploring the accuracy of self-reported maternal and newborn care in select studies from low and middle-income country settings: do respondent and facility characteristics affect measurement?
Source: BMC Pregnancy Childbirth. 2023 Jun 16;23:448. doi: 10.1186/s12884-023-05755-7 (PMC10273708; doi:10.1186/s12884-023-05755-7)
Supplement: Supplementary file 5 — Additional file 5. Univariate fixed effects model: self-reported ANC indicator accuracy by respondent and facility characteristics. [file 12884_2023_5755_MOESM5_ESM.docx]

Additional File 5. Univariate fixed effects model: self-reported ANC indicator accuracy by respondent and facility characteristics.

|  | Diagnostic Odds Ratio (DOR) (95%CI) by Predictors | | Significant Difference? | |
| --- | --- | --- | --- | --- |
|  |  |  |  |  |
| **Age Group** | Adolescents | Adults | | NA |
| Take weight | 144.6 (101, 207.1) | 68.1 (57.7, 80.4) | | NA |
| Blood pressure check | 58.1 (41.2, 82.1) | 51.7 (42.4, 63.1) | | NA |
| Abdominal exam | 61.4 (46.6, 80.8) | 40.4 (34.6, 47.2) | | NA |
| Anemia check/referral | 12.2 (10.2, 14.6) | 7.2 (6.6, 7.9) | | Y |
| Check fetal heart rate | 26.2 (21.4, 32.0) | 25.4 (22.5, 28.7) | | NA |
| Urine test | 14.7 (12.0, 18.0) | 17.0 (14.9, 19.4) | | NA |
| **Education** | **Less than primary completion** | **Primary completion or higher** | |  |
| Take weight | 55.9 (45.8, 68.3) | 110.5 (87.5, 139.5) | NA | |
| Blood pressure check | 36.7 (29.3, 45.9) | 79.0 (60.4, 103.4) | NA | |
| Abdominal exam | 32.6 (27.0, 39.3) | 55.7 (45.8, 67.7) | NA | |
| Anemia check/referral | 6.0 (5.4, 6.7) | 11.3 (10.0, 12.9) | Y | |
| Check fetal heart rate | 22.2 (19.2, 25.6) | 31.0 (26.6, 36.2) | Y | |
| Urine test | 12.6 (10.9, 14.7) | 20.5 (17.4, 24.2) | Y | |
| **Parity** | **First pregnancy** | **Prior pregnancy** |  | |
| Take weight | 73.3 (53.9, 99.8) | 41.7 (34.2, 50.8) | NA | |
| Blood pressure check | 60.2 (42.3, 85.7) | 25.7 (20.6, 32.0) | NA | |
| Abdominal exam | 22.0 (17.2, 28.1) | 22.3 (18.5, 27.0) | N | |
| Anemia check/referral | 6.2 (5.4, 7.1) | 6.5 (5.9, 7.2) | N | |
| Check fetal heart rate | 17.0 (14.2, 20.5) | 17.9 (15.5, 20.7) | N | |
| Urine test | 6.6 (5.4, 8.0) | 12.7 (10.9, 14.9) | Y | |
| **Right to care counseling** | **Non-voucher client** | **Voucher client** |  | |
| Take weight | 19.5 (15.8, 24.0) | 28.4 (18.5, 43.4) | NA | |
| Blood pressure check | 17.5 (13.5, 22.6) | 24.0 (16.3, 35.3) | NA | |
| Abdominal exam | 17.2 (14.4, 20.6) | 13.8 (9.2, 20.8) | N | |
| Anemia check/referral | 6.1 (5.4, 6.7) | 3.9 (3.3, 4.6) | Y | |
| Check fetal heart rate | 18.1 (15.7, 20.8) | 22.0 (17.1, 28.2) | NA | |
| Urine test | 8.3 (7.2, 9.7) | 26.5 (21.1, 33.2) | NA | |
| **Facility quality** | **Non-voucher facility** | **Voucher facility** |  | |
| Take weight | 95.1 (76.9, 117.7) | 64.3 (52.3, 79.1) | NA | |
| Blood pressure check | 50.0 (37.5, 66.7) | 51.5 (41.5, 64.0) | NA | |
| Abdominal exam | 36.4 (30.2, 43.9) | 53.5 (44.0, 65.1) | NA | |
| Anemia check/referral | 9.7 (8.6, 11.0) | 6.8 (6.2, 7.6) | Y | |
| Check fetal heart rate | 29.3 (25.1, 34.1) | 23.5 (20.5, 27.1) | N | |
| Urine test | 20.0 (16.4, 24.3) | 15.2 (13.2, 17.4) | N | |
| Notes: Comparison of estimates in grey have been suppressed due to low precision (margin of error $\geq$DOR ±5). * Denotes significant difference at p<0.05. Univariate fixed effects analysis was performed for antenatal care (ANC) indicators due to small sample size (N=3 studies) which assessed ANC. | | | | |
