# Supplementary material for: Exploring the accuracy of self-reported maternal and newborn care in select studies from low and middle-income country settings: do respondent and facility characteristics affect measurement?
Source: BMC Pregnancy Childbirth. 2023 Jun 16;23:448. doi: 10.1186/s12884-023-05755-7 (PMC10273708; doi:10.1186/s12884-023-05755-7)
Supplement: Supplementary file 6 — Additional file 6. Univariate fixed effects models: Self-reported PNC indicator accuracy by facility quality (non-voucher vs. voucher intervention facility). [file 12884_2023_5755_MOESM6_ESM.docx]

Additional File 6. Univariate fixed effects models: Self-reported PNC indicator accuracy by facility quality (non-voucher vs. voucher intervention facility).

|  | **Non-voucher facility** | **Voucher facility** |  |
| --- | --- | --- | --- |
| Indicator | Diagnostic Odds Ratio (DOR) (95%CI) | | Significant difference? |
| Blood pressure check | 24.2 (19.5, 30.1) | 13.1 (11.5, 19.6) | NA |
| Breast exam | 14.5 (12.1, 17.3) | 14.1 (12.1, 22.2) | NA |
| Abdominal exam | 18.9 (15.1, 23.5) | 16.8 (14.1, 28.1) | NA |
| Vaginal exam | 27.1 (21.6, 34.1) | 10.6 (8.8, 18.4) | NA |
| Anemia check/referral | 8.0 (9.4, 11.2) | 8.3 (7.3, 12.5) | N |
| Check/ask excessive bleeding | 8.3 (7.3, 9.5) | 5.0 (4.3, 7.7) | N |
| Discuss danger signs for mother | 4.0 (3.5, 4.7) | 4.2 (3.7, 6.1) | N |
| Discuss family planning | 7.4 (6.3, 8.6) | 7.1 (6.4, 10.0) | N |
| Discuss breast/infant feeding | 7.1 (6.0, 8.3) | 5.3 (4.8, 7.3) | N |
| Examine baby undressed | 5.1 (4.2, 6.2) | 1.8 (1.6, 2.4) | Y |
| Weigh baby | 7.9 (6.2, 9.9) | 8.0 (7.0, 11.9) | N |
| Given information on baby danger signs | 2.5 (2.2, 3.0) | 3.7 (3.2, 5.4) | Y |

Notes: Comparison of estimates in grey have been suppressed due to low precision (margin of error $\geq$DOR ±5). * Denotes significant difference at p<0.05. Univariate fixed effects analysis was performed for postnatal care indicators by facility quality due to small sample size (N=3 studies) which participated in the voucher intervention.
